# Supplementary material for: The causal relationship between the human gut microbiota and pyogenic arthritis: a Mendelian randomization study
Source: Front Cell Infect Microbiol. 2024 Nov 26;14:1452480. doi: 10.3389/fcimb.2024.1452480 (PMC11629706; doi:10.3389/fcimb.2024.1452480)
Supplement: Supplementary file 1 [file DataSheet1.zip › mendelian test/ebi-a-GCST90027506.csv_forest.pdf]

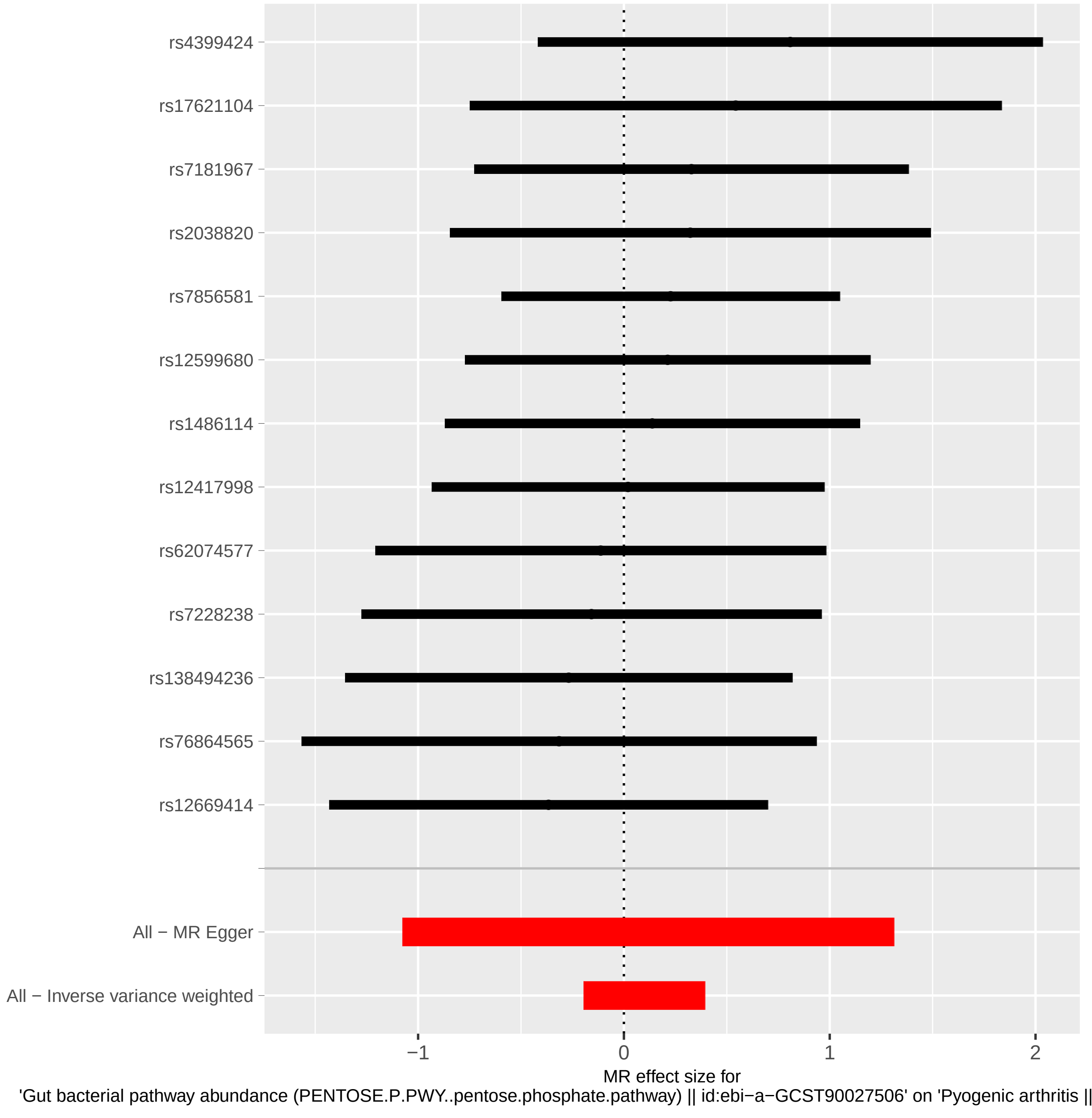
rs4399424
rs17621104
rs7181967
rs2038820
rs7856581
rs12599680
rs1486114
rs12417998
rs62074577
rs7228238
rs138494236
rs76864565
rs12669414
All − MR Egger
All − Inverse variance weighted
−1
0
1
2
MR effect size for
'Gut bacterial pathway abundance (PENTOSE.P.PWY..pentose.phosphate.pathway) || id:ebi−a−GCST90027506' on 'Pyogenic arthritis ||
